# S-Ketamine in the treatment of depressive emergencies: a cases series of patients in a suicidal crisis

**DOI:** 10.1192/j.eurpsy.2022.1446

**Published:** 2022-09-01

**Authors:** B. Baune, Z. Susam, V. Falcone, C. Knümann, P. Sarkheil, E. Kavakbasi

**Affiliations:** 1 Westfaelische-Wilhelms-University Muenster (WWU), Department Of Mental Health, Muenster, Germany; 2 University of Münster, Department Of Psychiatry, Münster, Germany

**Keywords:** s-ketamine, depression, suicidal ideation, emergency psychiatry

## Abstract

**Introduction:**

Psychiatric emergencies in Major Depressive Disorder (MDD) are characterised by multiple types of symptoms including risk of self-harm and suicidal ideation. S-ketamine intranasally (Spravato) has recently been shown to help alleviate symptoms during depressive emergencies. In this case-series, we detail the clinical effects and usability of S-ketamine applied intranasally in a psychiatric emergency setting.

**Objectives:**

To describe the effects of S-Ketamine on depressive crises associated with suicidality and self-harm in a psychiatric emergency setting.

**Methods:**

Patients with MDD in a psychiatric emergency were provided with intranasal S-Ketamine according to clinical indication in routine clinical care in a University inpatient setting. Clinical characteristics were assessed in a standardised manner and symptom measures were applied pre-and posttreatment. Experience with 10 patients is systematically described in this case-series.

**Results:**

Patients had a primary diagnosis of MDD accompanied by a variety of secondary psychiatric comorbidity. Among these 10 patients, the majority were female (70 %) and the mean age was 49.5 yrs (range 26-66). All cases were considered treatment resistant and suffered severe acute suicidal ideation. Across all cases, pre-treatment MADRS was 37 on average (range 20-47) indicating a severe form of MDD. High severity was confirmed in elevated BDI scores (pre-treatment 39). Post-treatment, MADRS scores were reduced to 18 on average, alongside BDI scores (mean 24). S-ketamine administration was well-tolerated and side effects such as dissociation were of short-lived duration.

**Conclusions:**

S-Ketamine intranasally can be safely and effectively administered in an acute psychiatric setting to treat psychiatric emergencies.

**Disclosure:**

BTB received honoraria for consultancy and presentations from AstraZeneca, Bristol-Myers Squibb, Lundbeck, Pfizer, Servier, Wyeth, LivaNova, Janssen, Novartis, Otsuka, Angelini.

